# The short-term efficacy of no-touch radiofrequency ablation in treating cirrhosis-based small hepatocellular carcinoma

**DOI:** 10.1186/s12885-019-5707-0

**Published:** 2019-05-27

**Authors:** Yuelong Chai, Kun Li, Chang Zhang, Shihan Chen, Kuansheng Ma

**Affiliations:** Institute of Hepatobiliary Surgery, Southwest Hospital, Third Military Medical University (Army Medical University), No. 30 Gaotanyan Main Street, Shapingba District, Chongqing, 400038 China

**Keywords:** No-touch, RFA, Small HCC, Cirrhosis, HBV

## Abstract

**Background:**

Radiofrequency ablation (RFA) for the treatment of small hepatocellular carcinoma (HCC) has a drawback of high recurrence rate. No-touch technique was developed to overcome it. However, it has barely been studied in Chinese populations. The aim of this study is to determine the safety and efficacy of no-touch RFA in the treatment of cirrhosis-based small HCC patients.

**Methods:**

A total of 130 patients of small HCC in Southwest Hospital were enrolled in this study, 46 cases treated by no-touch RFA and 84 cases by conventional RFA. Treatment complications and tumor-free survival rate and overall survival rate were compared and analyzed.

**Results:**

There were no significant differences in baseline confounding factors between the two groups. The ablation volume of no-touch RFA technique was significantly higher than conventional RFA (*P* = 0.002) but the remaining liver volume and treatment complications of the two techniques were the same (*P* = 0.702 and *P* = 0.269, respectively). Cox regression model revealed that conventional RFA was a predictive factor for short-term HCC recurrence (*P* = 0.041 for 2-year recurrence rate). Kaplan-Meier survival showed that tumor-free survival in no-touch group was significantly higher than conventional group (*P* = 0.047).

**Conclusions:**

Our data showed that no-touch RFA provided a higher short-term tumor-free survival rate than conventional RFA but was as safe as conventional RFA.

## Background

Liver cancer has become the tenth most common cancer of male in the United States as reported by American Cancer Society in 2017, but ranks the 5th of cancer death of all cancerous cases [1]. In China, this was even worse and liver cancer ranks the 4th of cancer incidence and 2nd of cancer death (estimated in 2011) [2]. The relatively low incidence and high mortality is a notable phenomenon in liver cancer, possibly due to a lack of curative treatment for advanced liver cancer [3].

Small hepatocellular carcinoma (HCC) is believed to be the curable stage of HCC and an effective intervention in small HCC is a practical way to decrease the high mortality of HCC [4]. Percutaneous thermal ablative techniques is a curative method for the treatment of small HCC except for hepatectomy and radiofrequency ablation (RFA) is first-line of this technique, as recommended by each liver study associations [5–8]. However, many HCC patients have to suffer from the second even third, fourth ablation because of the high recurrence rate. Therefore, multibipolar RFA was developed to overcome the shortcomings of conventional RFA, providing a larger necrosis margin and volume, and no-touch technique is one of the most frequent methods of multibipolar RFA [9]. Furthermore, according to the tumor-free principle, the no-touch technique was applied in RFA to avoid direct contact with liver tumor. Some researches have shown positive results. Compared with conventional RFA, no-touch RFA provided better primary RFA success and were as safe as conventional RFA while sustaining local tumor response [10, 11].

Although encouraging results were provided by previous studies, researches on the tumor-free survival rate and post-ablation complications of no-touch RFA in the treatment of HCC were still few, especially in the treatment of cirrhosis-based small HCC. In this research, we conducted a retrospectively case-control study to compare the tumor-free survival rate in cirrhosis-based small HCC patients between conventional RFA group and no-touch RFA group. We hope to add new evidence to the practice of no-touch RFA.

## Methods

### Study populations

From June 2015 to November 2015, a total of 130 HCC patients who underwent RFA at the Institute of Hepatobiliary Surgery, Southwest Hospital, Chongqing, China were retrospectively enrolled in this study. The inclusion criteria were: (1) single HCC nodule ≤3 cm by at least two enhanced image tests (enhanced CT/MRI or ultrasonography) as recommended by American Association for the Study of Liver Diseases [12, 13]; (2) cirrhotic basis diagnosed by enhanced image tests combined with physical examination and noninvasive serum markers as recommended by Japanese Society of Gastroenterology [14]; (3) first occurrence; (4) treated by conventional RFA or no-touch RFA; (5) Child-Pugh A/B; (6) follow-up up to 2 years; (7) without other types of cancer. Exclusion criteria included: (1) lost-to-follow-up or follow-up less than 2 years; (2) multiple HCC nodules or single HCC nodule> 3 cm; (3) combined with other cancers; (4) HCCs localized in “at risk” location (subcapsular, underdiaphragmatic, near gall bladder, etc.). Based on the different RFA methods, they were divided into two groups, conventional RFA group (C-RFA, 84 cases) and no-touch RFA group (NT-RFA, 46 cases). Manifestations of decompensation in cirrhosis include bleeding varices, hepatic encephalopathy (HE), hepato-renal syndrome and ascites.

### Radiofrequency ablation

A single operator with 10 years of experience in percutaneous ablation performed all procedures. Conventional RFA or no-touch RFA was chosen according to patients’ will after we made it understood to patients of all the strengths and shortcomings of the two techniques. General anesthesia was given before RFA treatment and all the procedures were conducted percutaneously under the guidance of real-time ultrasound. No-touch RFAs were performed as previously described by Seror et al. [9]. In short, puncture two or more electrode needles into the lesions adjacent to the tumor-free area (5 mm along the edge of the tumor) and by the guidance of ultrasound real-time scan, the scope of ablation include the tumor and 0.5 cm–1 cm liver tissue around the tumor edge to obtain a safe ablation area. The devices multipolar internally cooled-tip CelonProSurge™ (CelonPOWER System OLYMPUS Medical®) was used for operation. Conventional RFAs were performed likewise but only puncture one electrode needle into the center of the tumor. One day after RFA, an ultrasonography would be conducted. Patients who were full ablated would be discharged and patients with residuals would be conducted the second RFA at once.

### Follow-up and endpoint

One month after RFA, the treatment efficacy was evaluated in each patient by image tests (enhanced CT/MRI). For those who have residual cancerous margins would undertake the second RFA and assessed one month after was enrolled if still met the inclusion criteria. After first assessment, each patient undertook image examination every two months to monitor HCC local recurrence. The main endpoint of the study was the incidence of local recurrence after treatment. Local recurrence was defined as the appearance of tumor foci at the edge of the ablation zone. The secondary endpoints were intra-hepatic distant recurrence rate, disease-free survival and overall survival. Both survivals were calculated from date of initial treatment to date of disease recurrence or death.

### Statistical analysis

SPSS version 22.0 statistical software (IBM, USA) was applied for all statistical analysis and the graphs were constructed on the Prism version 7.00 (GraphPad Software Inc., USA). Each variable was represented as median with interquartile range. Student T-test or Mann-Whitney test was applied to compare the differences of continuous variables according to data distribution. Pearson Chi-square test and Fisher’s exact test was employed to evaluate statistical differences between categorical variables. For survival analysis, the tumor-free survival rate by patient groups was assessed with Kaplan-Meier analyses, and crude differences were calculated by log-rank test. Cox proportional hazard models with stepwise variable selection were used to calculate hazard ratios and 95% confidence intervals of HCC. Potent risk variables (*P* < 0.1) were entered into multivariate analyses, and independent risk factors were eliminated by backward selection method. A two-tailed *P* value less than 0.05 was defined to be statistically significant.

## Results

### Baseline patient characteristics

Baseline characteristics of all enrolled HCC patients were listed in Table 1, and data were presented as median with interquartile range. Baseline point was defined as the day or within 1 month of RFA. Except for tumor size, there were no significant differences in each parameters between NT-RFA and C-RFA group.

**Table 1:** Baseline characteristics of patients in two groups

	NT-RFA	C-RFA	*P* value
n	46	84	
Age, years	52.0 (43.0–58.0)	51.0 (45.0–59.0)	0.826
Gender, female	4 (8.7%)	6 (7.2%)	0.742*
BMI, kg/m^2^	23.9 (21.9–26.4)	23.6 (21.3–25.8)	0.622
Etiology			0.212*
HBV	44 (95.7%)	74 (88.1%)	
Others	2 (4.3%)	10 (11.9%)	
Cirrhotic basis			0.124
Compensated	32 (69.6%)	69 (82.1%)	
Uncompensated	14 (30.4%)	15 (17.9%)	
SLV, cm^3^	1405.0 (1341.0–1484.0)	1393.0 (1338.0–1454.0)	0.341
Child-Pugh			0.326*
A	44 (95.7%)	75 (89.3%)	
B	2 (4.3%)	9 (10.7%)	
Decompensated complications			0.102
Bleeding varices	5 (10.9%)	2 (2.4%)	
Hepato-renal syndrome	1 (2.2%)	0 (0)	
Hepatic encephalopathy	2 (4.3%)	1 (1.2%)	
Ascites	6 (13.0%)	12 (14.3%)	
Tumor size, mm	19.0 (15.0–22.0)	21.0 (16.0–26.0)	0.048
Near major vessels	12 (26.1%)	27 (32.1%)	0.551
Lab tests			
ALT, IU/l	30.5 (21.1–47.5)	31.6 (23.6–47.5)	0.450
TBIL, μmol/L	18.8 (14.6–23.2)	16.9 (13.9–23.1)	0.301
ALP, IU/L	107.0 (82.8–124.5)	90.0 (77.0–112.0)	0.075
TBA, μmol/L	5.7 (2.3–13.2)	7.6 (2.9–16.2)	0.270
PT, sec	11.9 (11.4–12.5)	12.0 (11.3–12.9)	0.327
Alb, g/L	44.7 (39.8–46.7)	44.8 (40.1–47.4)	0.634
PLT, 10^9/L	116.0 (88.0–163.3)	118.5 (78.5–147.5)	0.376
AFP, ng/ml	27.3 (4.4–224.6)	13.0 (3.3–105.8)	0.102

*Fisher’s exact test

*RFA* radio frequency ablation, *BMI* body mass index, *HBV* hepatitis B virus, *SLV* standard liver volume, *ALT* alanine aminotransferase, *TBIL* total bilirubin, *ALP* alkaline phosphatase, *TBA* total bile acid, *PT* prothrombin time, *Alb* albumin, *PLT* platelet count, *AFP* alpha-fetoprotein

SLV = 11.5 × body weight (kg) + 334 (Chengdu Formula)

As shown in the table, more than 90% HCC cases were male and more than 85% were HBV-related HCC cases, which is consistent with many previous reports. All patients were cirrhotic-based HCC cases but there were still 30.4 and 17.9% cases of uncompensated state in NT- and C-RFA group, respectively. Among them, ascites in 6 and 12 cases were detected by image test, respectively. Standard liver volume (SLV) by Chendu formula [15] was calculated in our study to assess the ablation risk combined with ablation volume. The median SLV was 1405.0 (interquartile range, IQR: 1341.0–1484.0) cm3 and 1393.0 (IQR: 1338.0–1454.0) cm3, respectively (*P* = 0.341). In this study, patients with small HCC with single nodule were enrolled and in both groups, the median diameters were less than 2 cm. Some laboratory tests were also tested and compared and there were no significant differences between two groups.

### Operation features

Operative characteristics were collected to compare the ablation risks between two RFA techniques, as shown in Table 2. Ablation duration in NT-RFA group (median: 8.2 min, IQR: 8.0–8.3 min) were significantly lower than C-RFA (median: 8.7 min, IQR: 8.2–8.8 min, *P* = 0.044), indicating the convenience of no-touch way. Length of stay was calculated from the day right after RFA and there were no significant differences between the two groups. In NT-RFA group, there were 3 patients undertaking 2 times of RFA therapy and 11 patients in C-RFA group, but there were no significant differences (*P* = 0.390). Interestingly, although the ablation area in NT-RFA group was significantly larger than C-RFA group (*P* = 0.002), there were no significant differences in the remaining liver volume (RLV) between these two groups (*P* = 0.702). Furthermore, as for complications and post-RFA total bilirubin (TBIL), there were no differences in two groups, either, even though the necrosis area by NT-RFA was much larger than C-RFA. As a result, No-touch technique would not increase the ablation risk, ablation failure or complications in RFA procedure as shown in our study.

**Table 2:** Operative characteristics of the two RFA

	NT-RFA (*n* = 46)	C- RFA (*n* = 84)	*P* value
Duration of ablation, min	8.2 (8.0–8.3)	8.7 (8.2–8.8)	0.044
Length of stay, day^a^	3.3 (3.0–3.7)	3.5 (3.2–3.7)	0.421
Ablation size, mm	45.5 (41.8–54.0)	41.0 (35.0–49.0)	0.002
RLV, mm^3^	1345.0 (1268.0–1444.0)	1359.0 (1263.0–1423.0)	0.702
More than one ablation	3 (6.5%)	11 (13.1%)	0.390
Ablation complications^b^	2 (4.8%)	10 (11.9%)	0.269***
Ascites	2 (4.8%)	6 (7.1%)	
Sepsis	0 (0)	2 (2.4%)	
Active bleeding	0 (0)	2 (2.4%)	
Post-TBIlL(2 h), μmol/L	24.2 (20.8–34.4)	26.6 (18.3–35.2)	0.862
Local recurrence	1 (2.2%)	13 (15.5%)	0.042***
Distant recurrence	4 (8.7%)	8 (9.5%)	0.872

^a^The time before RFA was not included for comparison

^b^According to the society of interventional radiology classification

***Fisher’s exact test

*RFA* radio frequency ablation, *RLV* remaining liver volume, *TBIL* total bilirubin

Ablation size was evaluated by ultrasonography after RFA

RLV=SLV-ablation volume, ablation volume was calculated based on the formula of sphere volume

### Operative outcomes

One month after RFA procedure, the first examination would be conducted to evaluate RFA efficacy. Image examination was used to assess if there were still cancerous margins or untreated nodules after ablation and laboratory tests were used to evaluate treatment safety and therapeutic efficacy. Table 3 gave the results. In NT-RFA group, there were no significant differences in alkaline phosphatase (ALP) levels before (median: 101.0 IU/L, IQR: 81.8–118.3 IU/L) and after (median: 100.0 IU/L, IQR: 86.8–138.8 IU/L) ablation (*P* = 0.384), but in C-RFA group, one month after RFA, the ALP level significantly elevated (median level from 90.0 IU/L to 110.0 IU/L, *P* < 0.001). In addition, levels of platelet counts (PLT), alpha-fetoprotein significantly decreased after ablation in both NT-RFA group and C-RFA group, suggesting an encouraging therapeutic efficacy of RFA. However, levels of aspartate aminotransferase (AST) significantly increased after ablation in both groups, suggesting the damage to liver still exist after one month. Levels of other indexes recover to the normal range one month after ablation. Interestingly, the local recurrence rate of NT-RFA was significantly lower than C-RFA (2.2% vs 15.5%, *P* = 0.042), but there was no significant difference in distant recurrence (8.7% vs 9.5%, *P* = 0.872), indicating that the NT-RFA provide a bigger ablation range and performed better than C-RFA in destroying localized tumor.

**Table 3:** Post-operative outcomes of HCC patients 1 month after RFA

	NT-RFA (*n* = 46)			C-RFA (*n* = 84)		
	Pre-RFA	Post-RFA	*P* value	Pre-RFA	Post-RFA	*P* value
ALT, IU/L	30.4 (21.1–46.3)	34.8 (22.8–69.4)	0.100	31.7 (23.8–46.5)	34.0 (25.0–53.9)	0.941
TBIL, μmol/L	18.8 (14.6–22.7)	18.4 (15.5–25.5)	0.419	17.3 (12.6–21.8)	21.1 (14.1–29.6)	0.233
ALP, IU/L	101.0 (81.8–118.3)	100.0 (86.8–138.8)	0.384	90.0 (77.0–112.0)	110.0 (87.0–145.0)	< 0.001
TBA, μmol/L	5.7 (2.5–11.4)	8.1 (2.9–20.0)	0.233	7.6 (2.9–16.2)	5.6 (3.1–13.6)	0.304
AST, IU/L	31.9 (27.0–44.6)	37.9 (30.8–50.7)	< 0.001	34.9 (28.4–45.2)	37.7 (31.2–50.0)	0.363
Alb, g/L	44.7 (40.1–46.7)	43.3 (37.9–47.2)	0.149	44.8 (40.1–47.4)	42.5 (30.7–45.8)	0.045
PLT, 10^9/L	122.5 (91.8–163.3)	104.5 (74.0–138.0)	< 0.001	118.5 (78.5–147.5)	103.0 (77.0–150.5)	0.112
AFP, ng/mL	22.1 (4.3–224.6)	5.0 (3.0–13.7)	< 0.001	13.0 (3.3–105.8)	4.9 (2.8–15.2)	< 0.001

*RFA* radio frequency ablation, *ALT* alanine aminotransferase, *TBIL* total bilirubin, *ALP* alkaline phosphatase, *TBA* total bile acid, *AST* aspartate aminotransferase, *Alb* albumin, *PLT* platelet count, *AFP* alpha-fetoprotein

### Analysis of short-term overall survival and tumor-free survival rate

After RFA, the recurrence rate and overall survival rate of all HCC patients would be assessed for 24 months to compare the differences and to find the predictive factors. Table 4 showed the predictive factors of 2-year recurrence rate. Female, age < 40 years, body mass index (BMI) < 24 kg/m^2^, compensated cirrhotic basis, AST-to-Platelet Ratio Index (APRI) < 0.5, no ascites, tumor size≤20 mm, alanine aminotransferase (ALT) ≤40 IU/L, AST ≤40 IU/L, prothrombin time (PT) ≤13 s, ALP ≤110 IU/L, total bile acid (TBA) ≤10 μmol/L, albumin-bilirubin (ALBI) grade 1, PLT ≥100*10^9/L, alpha-fetoprotein (AFP) ≤20 ng/ml, Child-Pugh A and no-touch RFA were set as reference. In the Cox regression model, AST > 40 IU/L and conventional RFA were predictive factors in univariate analysis and in multivariate analysis, conventional RFA was the only predictive factors of 2-year recurrence (Hazard Ratio, HR = 2.11; 95% confidence interval, 95% CI: 1.16–4.43; *P* = 0.041). Univariate analysis and multivariate analysis were also conducted for overall survival, however, no factor was significantly associated with the overall survival. This maybe be due to the relatively short period of follow-up time and limited patients enrolled.

**Table 4:** Predictive factors of 2-year recurrence rate

Variables	N	Univariate analysis		Multivariate analysis	
		HR (95% CI)	*P* value	HR (95% CI)	*P* value
Gender, male	120	1.46 (0.19–10.99)	0.713	1.44 (0.19–10.73)	0.725
Age (years)					
< 40(ref)	14				
40–60	91	1.51 (0.65–3.82)	0.633		
> =60	25	2.70 (0.55–8.15)	0.221		
BMI (kg/m^2^), > = 24	61	1.71 (0.76–3.84)	0.193		
Cirrhotic state, Uncompensated	32	1.09 (0.44–2.72)	0.849		
APRI					
< 0.5(ref)	32				
0.5–1.5	64	0.72 (0.22–2.39)	0.590		
> =1.5	34	1.19 (0.46–3.07)	0.723		
Pre-treatment ascites	18	4.83 (0.65–35.77)	0.124		
Pre-treatment EGV	7	1.22 (0.39–2.16)	0.256		
Tumor size (mm), > 20	64	1.13 (0.52–2.45)	0.756		
ALT (IU/L), > 40	47	1.29 (0.56–2.96)	0.551		
AST (IU/L), > 40	45	2.28 (0.86–6.05)	0.098	2.25 (0.85–5.98)	0.104
PT (sec), > 13	23	1.29 (0.38–4.38)	0.683		
ALP (IU/L), > 110	41	1.84 (0.73–4.63)	0.194		
TBA (μmol/L), > 10	48	1.11 (0.50–2.49)	0.799		
ALBI grade					
1(ref)	98				
2	32	1.04 (0.42–2.61)	0.931		
3	0				
PLT(10^9/L), < 100	53	1.03 (0.47–2.29)	0.939		
AFP (ng/ml), > 20	59	1.08 (0.50–2.35)	0.849		
Child-Pugh, B	11	1.62 (0.62–4.69)	0.214		
Conventional RFA	84	2.12 (1.16–4.62)	0.027	2.11 (1.16–4.43)	0.041

*RFA* radio frequency ablation, *BMI* body mass index, *APRI* Aspartate aminotransferase-to-Platelet Ratio Index, *ALT* alanine aminotransferase, *AST* aspartate aminotransferase, *PT* Prothrombin time, *ALP* alkaline phosphatase, *EGV* esophago-gastric Varices, *TBA* total bile acid, *ALBI* albumin-bilirubin grade for HCC, *PLT* platelet count, *AFP* alpha-fetoprotein

**Fig. 1:**
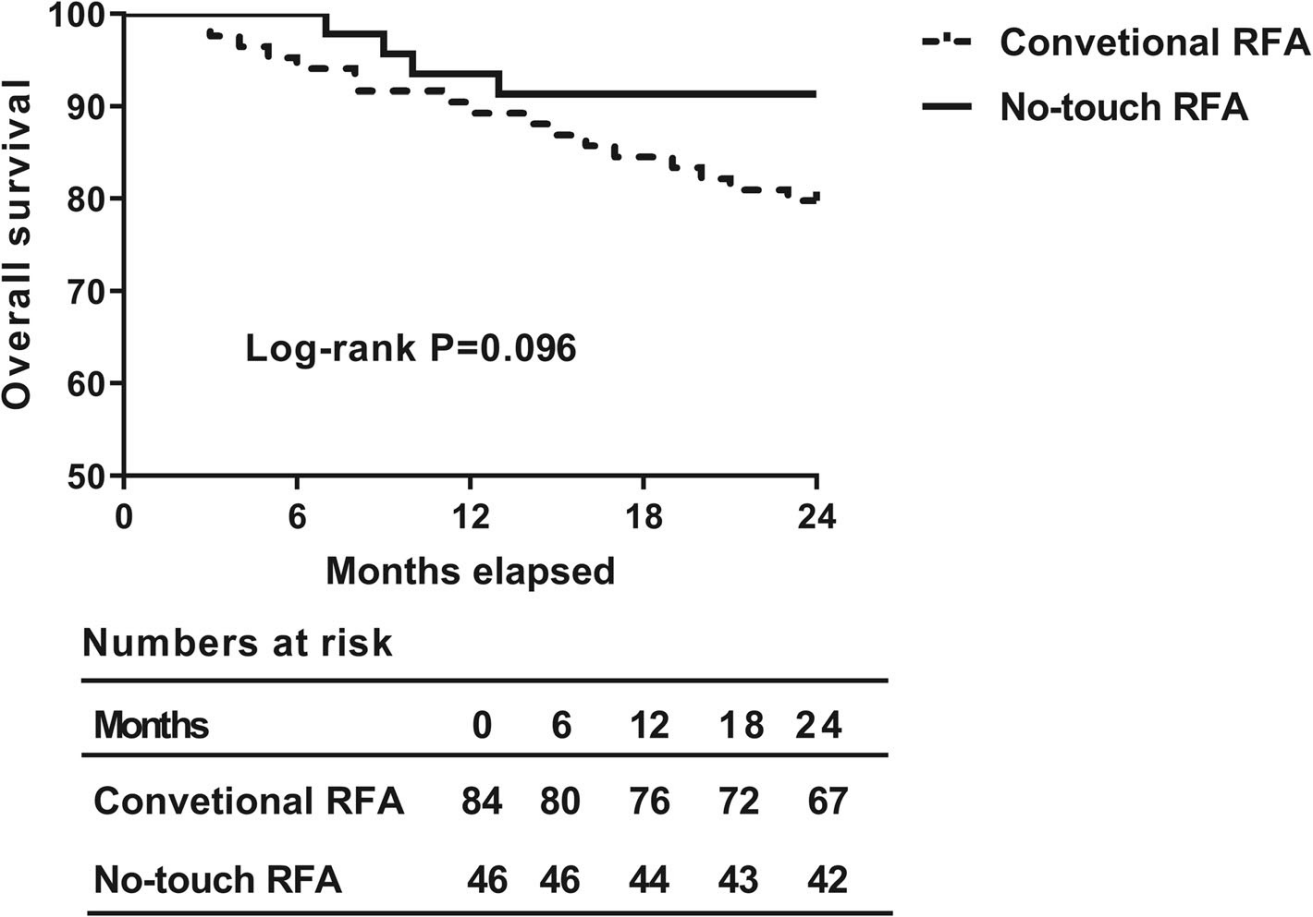
The overall survival curves of different RFA techniques. The table shows the exact number of at-risk patients at each time point

**Fig. 2:**
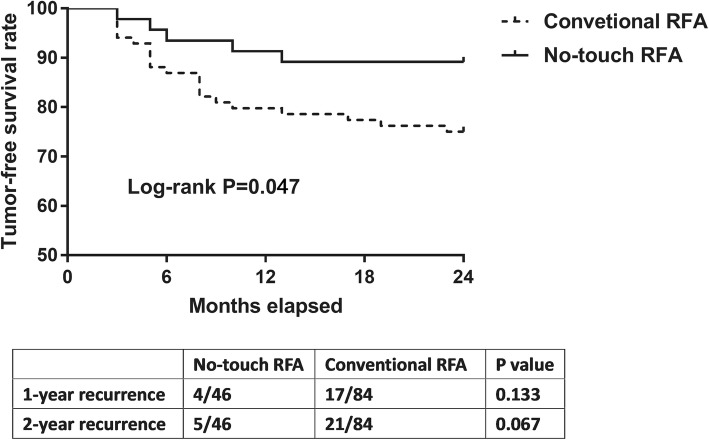
Kaplan-Meier tumor-free survival curves divided by different RFA techniques. The table shows the recurrence cases 1- and 2- year after RFA. P value was calculated by Pearson Chi-square test or Fisher’s exact test

Figure 1 showed the overall survival of HCC patients by Kaplan-Meier. Although the overall survival rate in NT-RFA group was higher than in C-RFA group, there was no significant difference between the two groups (Log-rank *P* = 0.096). However, things are different for tumor-free survival rate. Figure 2 showed the tumor-free survival of HCC patients divided by different RFA methods. The tumor-free survival rate in NT-RFA group was higher than C-RFA group in 2-year follow-up (Log-rank *P* = 0.047). After one year, 4 patients recurred in NT-RFA group and 17 in C-RFA group (*P* = 0.133). At the end of follow-up, 5 cases recurred in NT-RFA group and 21 recurred in C-RFA group (*P* = 0.067).

## Discussion

Although RFA has been listed as the curative method for the treatment of small HCC, many studied have reported a 10–30% recurrence rate after RFA treatment [16–20]. A research from Japan enrolling 7185 cases of small HCC (< 3 cm) showed a 55.4% recurrence rate in 2 years’ time compared with resection group [21]. A study from China reported a recurrence rate up to 72.7% [22]. The high recurrence rate was partly due to the untreated cancerous margins and unobserved nodules [23]. In an attempt to increase the ablation volume and then eliminate the cancerous margins in RFA treatment of small HCC, the no-touch technique was developed.

Principally, no-touch RFA has the following strengths: Firstly, no-touch RFA could reach maximum energy deposition around the tumor to kill cancer cell to the full and at the same time guarantee a safe margin. However, conventional RFA transfers heat from the center to the periphery, which may result in the phenomenon of “heat-sink” and reduce the efficacy [24, 25]. Secondly, some report found that multipolar technique could effectively reduce the newly-developed tumor vessels around the margin. Thirdly, as its name indicated, no-touch technique avoid touching the tumor and cause less blood loss so as to reduce the risk of tumor plantation and metastasis [26]. However, in the aspect of ablation safety, multipolar adds the puncture times and may increase the operational risk. So in clinical practice, irrigation of saline while ablation was applied to protect vital vessels and organs to reduce complications. In a word, the no-touch technique is the tendency of RFA but the indications and contraindications still remain discussion.

After the first case of no-touch been reported [27], some studies have focused on the efficacy of this new technique on the treatment of HCC. Results from an experimental animal study showed that the puncture localization, puncture distribution and prognosis between nodules less than 2.5 cm and more than 2.5 cm were different [28]. Three years later, they evaluated the usefulness of no-touch RFA in preventing intra-subsegmental tumor recurrence for HCC patients, concluding that no-touch pincer ablation procedure has the potential to prevent intra-subsegmental recurrence after RFA for patients with HCC [11]. In 2014, Olivier Seror et al. reported the histopathologic research of small HCC after RFA, discovering that no-touch RFA could improve the rate of complete necrosis compared with conventional RFA. Two-year after this histopathologic analysis, the same team gave the long-term results of 108 HCC patients after no-touch RFA, showing that 3- and 5-year local and overall tumor-free survival were 96, 94, 52, and 32%, respectively [25]. Besides, results from Premal A. Patel et al. showed one case of recurrence out of 8 liver cancers after no-touch ablation in 244 days [29]. Our study compared the 2-year tumor-free survival rate of no-touch RFA and conventional RFA in the treatment of small HCC. The result showed that 1-year rate was 8.7% of the no-touch group and 20.3% of the conventional group, and the 2-year rate was 10.9% of the no-touch group and 25.4% of the conventional group, which was consistent with these researches.

Studies on the comparison between no-touch RFA and conventional RFA in the treatment of small HCC were quite few. Arnaud Hocquelet et al. for the first time reported the multicenter results on the comparison of no-touch RFA and monopolar RFA for small HCC [10]. The coarsened exact matching was used to eliminate confounding factors. However, they mainly focused on the safety and complications of the two techniques and concluded that there was no significant difference on the overall survival rate of the two techniques but no-touch technique provided better primary RF success. Interestingly, in our study, no-touch RFA was the independent predictive factor for a low 2-year tumor-free survival rate (*P* = 0.047). This phenomenon indicated that no-touch RFA could improve the short-term tumor-free survival rate so as to reduce the frequency of RFA and financial burdens. In a word, no-touch RFA was a proper choice for a better primary outcome.

In this study, cirrhosis-based HCC was selected as the object. Most of them were HBV-related cases, which was different from previous studies. As far as we know, this was the first report about the efficacy of no-touch RFA on the treatment of HBV-related small HCC. However, some limitations exist. As we all know, in HBV-based HCC patients, viral factors was closely associated with HCC occurrence and recurrence. However, in this retrospective study, many data were not measured at baseline point, especially for viral indexes, so it’s a pity that we could not analyze the influence of this part of information on the HCC recurrence. Besides, this study was a retrospective non-randomized research and the bias exist. Moreover, the sample size was relatively small and follow-up time was short. When no-touch RFA was operated at the beginning, patients with Child-Pugh A or smaller HCC were selected. And for risky operations, like HCC near main vessels and gallbladder, we often chose conventional RFA, which caused selection bias. To reduce the selection bias to the minimum, all the cases with dangerous locations were excluded in this study both by NT-RFA and C-RFA, even when the operator was skilled enough to perform operations at dangerous location later. Except for this, the operating methods of the other enrolled patients were selected randomly to minimize the bias. Nevertheless, the results of this study should be further confirmed by prospective multicenter randomized trials of large samples.

## Conclusion

In conclusion, this case-matched study on the cirrhosis-based small HCC showed that no-touch RFA was a proper treatment for HBV-related small HCC and compared with conventional RFA, no-touch RFA provided a higher short-term tumor-free survival rate after treatment and as safe as conventional RFA.

## Abbreviations

AFP: Alpha-fetoprotein
ALBI: Albumin-bilirubin
ALP: Alkaline phosphatase
ALT: Alanine aminotransferase
APRI: AST-to-Platelet Ratio Index
AST: Aspartate aminotransferase
BMI: Body mass index
CI: Confidence interval
HCC: Hepatocellular carcinoma
HE: Hepatic encephalopathy
HR: Hazard ratio
IQR: Interquartile range
NT: No-touch
PLT: Platelet counts
PT: Prothrombin time
RFA: Radiofrequency ablation
RLV: Remaining liver volume
SLV: Standard liver volume
TBA: Total bile acid
TBIL: Total bilirubin
